# Impact of Physical Activity on All-Cause Mortality According to Specific Cardiovascular Disease

**DOI:** 10.3389/fcvm.2022.811058

**Published:** 2022-02-04

**Authors:** Moon-Hyun Kim, Jung-Hoon Sung, Moo-Nyun Jin, Eunsun Jang, Hee Tae Yu, Tae-Hoon Kim, Hui-Nam Pak, Moon-Hyoung Lee, Gregory Y. H. Lip, Pil-Sung Yang, Boyoung Joung

**Affiliations:** ^1^Division of Cardiology, Department of Internal Medicine, Yonsei University College of Medicine, Seoul, South Korea; ^2^Division of Cardiology, CHA Bundang Medical Center, CHA University, Seongnam, South Korea; ^3^Division of Cardiology, Department of Internal Medicine, Sangye Paik Hospital, Inje University College of Medicine, Seoul, South Korea; ^4^Liverpool Centre for Cardiovascular Science, University of Liverpool, Liverpool Heart and Chest Hospital, Liverpool, United Kingdom

**Keywords:** physical activity, all-cause mortality, cardiovascular disease, senior adults, stroke, heart failure

## Abstract

**Background:**

Patients with cardiovascular disease (CVD) tend to have higher mortality rates and reduced physical activity (PA). We aimed to evaluate the effect of PA on mortality in older adults with specific CVD.

**Methods:**

We enrolled 68,223 participants (*n* = 23,871 with CVD, *n* = 44,352 without CVD) aged ≥65 years with available physical activity data between 2005 and 2012 from the Korean National Health Insurance Service of Korea-Senior database. CVD was defined as a history of ischemic stroke, transient ischemic attack, heart failure, myocardial infarction, and peripheral artery disease.

**Results:**

Patients with CVD were older than those without CVD. Compared with the sedentary group, the physically active groups with and without CVD had a lower incidence and risk of all-cause death during a median follow up period of 42 (interquartile range 30–51) months. A 500 metabolic equivalent task-min/week increase in PA resulted in an 11% and 16% reduction in the risk of mortality in the non-CVD and CVD groups, respectively. With regard to specific CVDs, the risk of mortality progressively reduced with increasing PA in patients with heart failure or myocardial infarction. However, the reduction reached a plateau in patients with stroke or peripheral artery disease, but was significantly greater in patients with stroke (20% vs. without stoke, 11%, P_int_ = 0.006) or heart failure (13% vs. without heart failure, 11%; P_int_ = 0.045)

**Conclusions:**

PA was associated with a reduced risk of all-cause mortality in older adults with and without CVD. The benefits of PA in patients with CVD, especially patients with stroke or heart failure, were greater than those without.

## Introduction

The inverse relationship between physical activity and mortality has been proven in previous studies (1, 2). Current guidelines recommend that adults should perform at least 150–300 min/week of moderate-intensity physical activity, 75–150 min/week of vigorous physical activity, or 500–1,000 metabolic equivalent task (MET)-min/week of moderate-to-vigorous physical activity (3, 4). Satisfying recommended guidelines for physical activity is associated with a reduced risk of all-cause mortality, cardiovascular mortality, morbidity, and frailty compared with being inactive (5, 6).

The existing evidence on the dose relationship between exercise and outcomes has mostly been obtained from studies on healthy people (7–9). Individuals with cardiovascular disease (CVD) have higher mortality and morbidity, but also tend to have a sedentary lifestyle and less physical activity than those without CVD (10). While physical activity is generally recommended for secondary CVD prevention according to current guidelines, there are limited data regarding the relationship between physical activity and mortality, specifically among patients with pre-existing CVD (11–13). Recently, Jeong et al. reported that the benefit in the secondary prevention group was greater than that in the primary prevention group: every 500 MET-min/week increase in physical activity resulted in a 14 and 7% reduction in the risk of mortality in the secondary and primary prevention groups, respectively (interaction *P* < 0.001). However, the benefits of physical activity have not been precisely elucidated, especially in elderly patients with CVD (14).

In this study, we analyzed a population-based cohort to investigate the effect of physical activity on mortality in elderly populations with or without CVD. The aims of this study were as follows: (i) to identify the relationship between physical activity and mortality among elderly patients with and without CVD and (ii) to compare the associations between physical activity and mortality according to specific CVD.

## Methods

### Study Population

Data were collected from the National Health Insurance Service of Korea (NHIS)-Senior database, which includes data for 5,58,147 individuals recruited by 10% simple random sampling from a total of 5.5 million populations aged ≥60 years in the National Health Information Database (15, 16). Individuals covered by the insurance system undergo a general health screening every 2 years. The NHIS-Senior database includes data on the following parameters: sociodemographic and socioeconomic information, health check-up examinations, insurance status, and records of participants' medical histories. This study was approved by the Institutional Review Board of the Yonsei University Health System (4-2021-0894). The study complied with the requirements of the Declaration of Helsinki, and the need for informed consent was waived.

From the Korean NHIS-Senior database, 68,223 participants aged ≥65 years with available physical activity data between 2005 and 2012 were enrolled in this study and followed up until December 2014. Each individual's claims records were reviewed for a history of CVD from 2002 until the date of the health check-up. Participants with prior myocardial infarction, peripheral artery disease, other vascular diseases, ischemic/hemorrhagic stroke, transient ischemic attack, and heart failure were considered to have CVD. Patients with one or more of the CVD were based on the first diagnosed CVD. A flow chart of the study population enrollment ant analysis is presented in Supplementary Figure 1.

Information on comorbid conditions was identified from the International Classification of Disease-10 codes, and prescription medications before the index date (Supplementary Table 1). In order to ensure diagnostic accuracy, participants were considered to have comorbidities when the condition was a discharge diagnosis or was confirmed at least twice in an outpatient setting, as in our previous studies (15–24).

### Physical Activity Level Assessment

The leisure-time physical activity level was assessed using self-reported intensity and frequency of exercise via structured questionnaires using a 7-day recall method (25). The survey included three questions that assessed the usual frequency (days per week) of (i) vigorous physical activity for at least 20 min, (ii) moderate physical activity for at least 30 min, and (iii) light physical activity for at least 30 min. Vigorous physical activity was defined as intense exercise that caused severe shortness of breath, such as running and cycling at high speed. Moderate physical activity was defined as exercise that caused mild shortness of breath, such as brisk walking and cycling at usual speed. Light physical activity was defined as walking at a slow or leisurely pace. Completion of physical activity level assessment was performed during health check-up between 2005 and 2012 and followed up until December 2014.

Ratings of 3.3, 4.0, and 8.0 METs were assigned for light, moderate, and vigorous physical activity, respectively (20). The physical activity-related energy expenditure (MET-min/week) was calculated by summing the products of frequency, intensity, and duration of light, moderate, and vigorous physical activity. The participants were stratified on the basis of their weekly total physical activity levels as follows: (1) sedentary group: no leisure-time physical activity beyond basic movements; (2) insufficiently active group: energy expenditure between 1 and 499 MET-min/week; (3) active group: energy expenditure between 500 and 999 MET-min/week; (4) highly active: energy expenditure between 1,000 and 1,499 MET-min/week; and (5) very highly active group: energy expenditure ≥1,500 MET-min/week according guidelines and previous studies (3, 4, 20).

### Outcomes

The endpoint was all-cause mortality. Using unique personal identification numbers, information on death (date and cause of death) was confirmed from the National Population Registry of the Korea National Statistical Office, in which deaths are centrally registered on the basis of death certificates (15–24). The NHIS and National Statistical Office are national agencies serving the entire Korean population, so this approach provides a complete event check. We also analyzed cause-specific mortality based on the causes of death confirmed by the Korea National Statistical Office.

### Statistical Analysis

Descriptive statistics were used to characterize baseline characteristics and comorbidities. Categorical variables are reported as frequencies (percentages). Continuous variables are expressed as medians with interquartile ranges. Categorical variables were compared using Fisher's exact test or Pearson's chi-square test, and continuous variables were compared using Student's *t*-test.

Incidence rates of mortality were calculated by dividing the number of events by person-time at risk and presented as the rate per 1,000 person-years. We analyzed the hazard ratios and 95% confidence intervals or mortality according to the physical activity level. Competing risk regression was performed using the Fine-Gray sub distribution hazard model, with mortality as a competing risk for mortality events. Multivariable regression models were constructed by adjusting for age, sex, body mass index, smoking, alcohol consumption, hypertension, diabetes mellitus, dyslipidemia, chronic kidney disease, chronic obstructive pulmonary disease, osteoporosis, malignancy, hospital frailty risk score, and Charlson comorbidity index score. We used cubic spline curves to examine the effects of continuous values of physical activity (0 MET-min/week as reference) on all-cause mortality. We conducted subgroup analyses for the primary outcome stratified by age, sex, body mass index, and other baseline comorbidities.

All tests were two-tailed, and statistical significance was set at *p* < 0.05. Statistical analyses were conducted using R programming version 4.0.3 (The R Foundation for Statistical Computing, Vienna, Austria).

## Results

The baseline characteristics of the 68,223 study subjects are shown in Table 1. The median age of the participants was 73.6 years, and 38.8% were men. In the study population, 35.0% of participants had CVD (*n* = 23,871) and the remaining 65.0% (*n* = 44,352) had no history of CVD. In the CVD group, participants were older (74.7 vs. 73.0 years) and the proportion of men was lower (34.8 vs. 41.0%) than that in the non-CVD group (*P* < 0.001).

**Table 1:** Baseline characteristics according to cardiovascular disease.

**Variables**	**Total (*N* = 68,223)**	**Cardiovascular disease (*N* = 23,871)**	**No cardiovascular disease (*N* = 44,352)**	***P* value**
**Demographic**
Age, years	73.6 ± 5.7	74.7 ± 6.0	73.0 ± 5.4	<0.001
Male	26,483 (38.8)	8,299 (34.8)	18,184 (41.0)	<0.001
Body mass index	23.8 ± 3.4	24.1 ± 3.5	23.6 ± 3.3	<0.001
Waist	83.1 ± 8.9	84.0 ± 9.2	82.6 ± 8.8	<0.001
Systolic blood pressure	131.3 ± 17.1	131.2 ± 17.2	131.3 ± 17.0	0.430
Diastolic blood pressure	78.3 ± 10.5	77.9 ± 10.6	78.4 ± 10.4	<0.001
Smoking	16,718 (24.5)	5,136 (21.5)	11,582 (26.1)	<0.001
Alcohol	13,431 (19.7)	3,430 (14.4)	10,001 (22.5)	<0.001
**Income status**				<0.001
Low	20,927 (30.7)	7,214 (30.2)	13,713 (30.9)	-
Mid	15,018 (22.0)	5,110 (21.4)	9,908 (22.3)	
High	32,278 (47.3)	11,547 (48.4)	20,731 (46.7)	
**Risk scores**
Hospitality frailty risk score	2.0 ± 4.2	3.6 ± 5.8	1.1 ± 2.5	<0.001
Charlson comorbidity index	3.2 ± 2.7	5.0 ± 2.8	2.2 ± 2.2	<0.001
**Pre-existing non-cardiovascular disease**
Hypertension	41,726 (61.2)	20,279 (85.0)	21,447 (48.4)	<0.001
Diabetes mellitus	14,767 (21.6)	7,430 (31.1)	7,337 (16.5)	<0.001
Dyslipidemia	35,048 (51.4)	17,202 (72.1)	17,846 (40.2)	<0.001
Chronic kidney disease	1,937 (2.8)	1,236 (5.2)	701 (1.6)	<0.001
COPD	8,505 (12.5)	4,302 (18.0)	4,203 (9.5)	<0.001
Malignancy	11,141 (16.3)	4,881 (20.4)	6,260 (14.1)	<0.001
Osteoporosis	28,476 (41.7)	12,150 (50.9)	16,326 (36.8)	<0.001
**Pre-existing cardiovascular disease**
Heart failure	8,982 (13.2)	8,982 (37.6)	-	-
Ischemic stroke or TIA	13,172 (19.3)	13,172 (55.2)		
Previous MI	3,126 (4.6)	3,126 (13.1)		
Peripheral artery disease	6,623 (9.7)	6,623 (27.7)		
**Laboratory findings**
Fasting blood glucose	106.1 ± 31.6	108.5 ± 34.5	104.8 ± 29.8	<0.001
Total cholesterol	196.1 ± 40.5	190.7 ± 41.8	199.0 ± 39.5	<0.001
Triglyceride	140.0 ± 80.9	142.4 ± 80.9	138.8 ± 80.9	<0.001
LDL-cholesterol	116.0 ± 37.9	111.4 ± 38.6	118.5 ± 37.3	<0.001
HDL-cholesterol	53.1 ± 25.4	51.6 ± 23.5	53.9 ± 26.4	<0.001
AST	26.5 ± 19.1	26.1 ± 18.6	26.8 ± 19.4	<0.001
ALT	21.9 ± 18.0	21.6 ± 17.3	22.1 ± 18.4	<0.001
Gamma-GT	33.6 ± 52.8	32.9 ± 46.6	34.0 ± 55.8	<0.001
Serum creatinine	1.0 ± 1.0	1.1 ± 1.2	1.0 ± 0.8	0.004
eGFR	71.2 ± 17.8	67.9 ± 19.1	73.0 ± 16.8	<0.001

* *Values are presented as mean ± standard deviation, median (Q1, Q3, quartiles [25th and 75th percentiles]), or %*.

*COPD, chronic obstructive pulmonary disease; TIA, transient ischemic attack; MI, myocardial infarction; LDL, low-density lipoprotein; HDL, high-density lipoprotein; AST, aspartate aminotransferase; ALT, alanine aminotransferase; Gamma-GT, gamma-glutamyl transpeptidase; eGFR, estimated glomerular filtration rate*.

**Figure 1:**
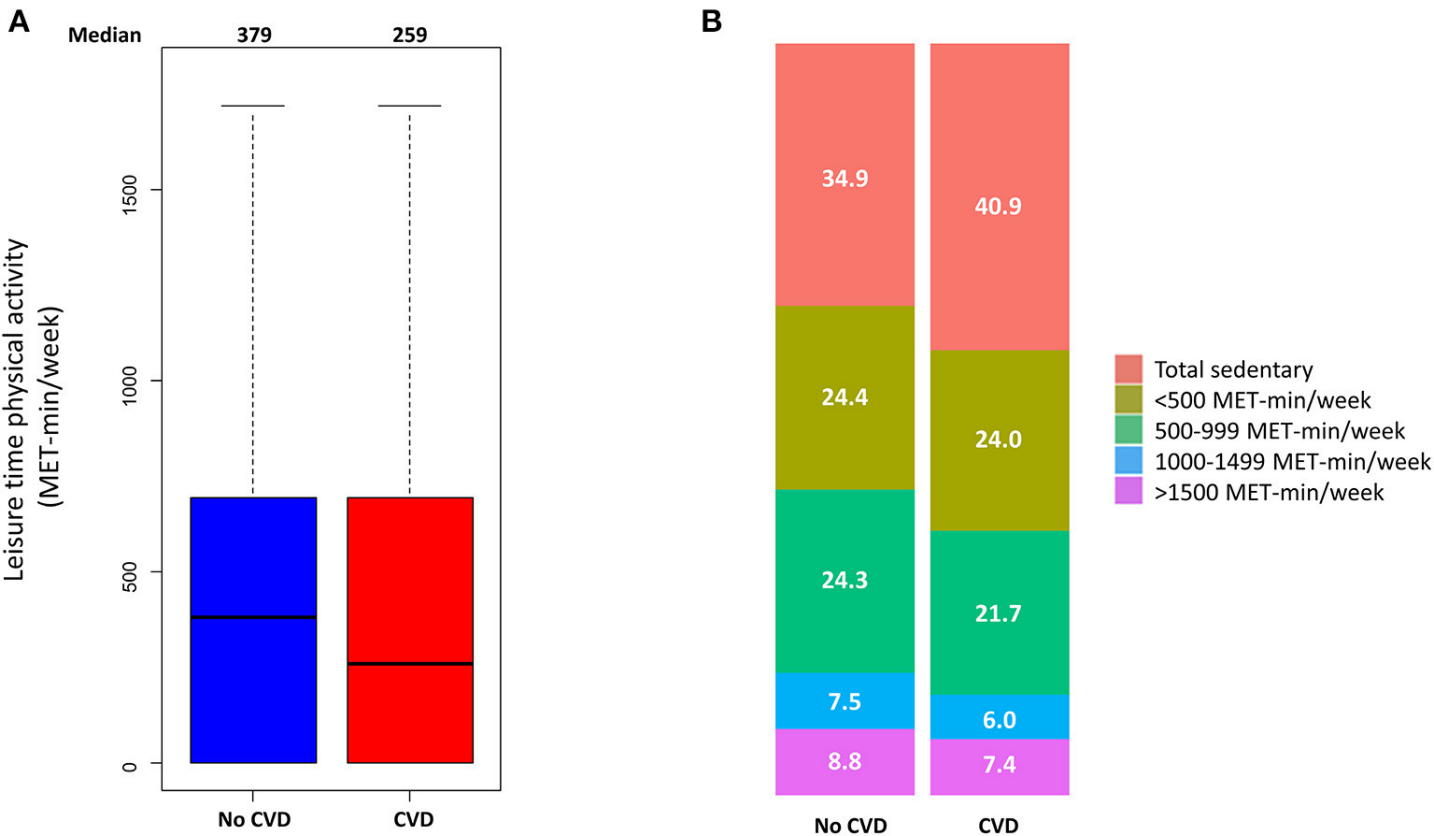
Physical activity of participants with or without cardiovascular disease (CVD). **(A)** Box and whiskers plot for physical activity, **(B)** box plot for the proportions of physical activity classified into five categories.

The range of physical activity level of the study population was 0-2653 MET-min/week. The median physical activity levels were 259 and 379 MET-min/week in the CVD and non-CVD groups, respectively (Figure 1). Participants with CVD were less physically active than those without CVD (*P* < 0.001). The proportion of sedentary participants was higher in the CVD group than in the non-CVD group (40.9 and 34.9%, respectively, *P* < 0.001) (Figure 1). Supplementary Figure 2 shows the proportions of participants with specific CVDs. There were differences in the median physical activity according to the history of stroke or heart failure, with no significant differences in physical activity in patients with peripheral artery disease or myocardial infarction.

Baseline characteristics of the whole study population, CVD group, and non-CVD group according to leisure-time physical activity are shown in Supplementary Tables 2–4, respectively.

### Impact of Physical Activity on All-Cause Mortality According to CVD

**Figure 2:**
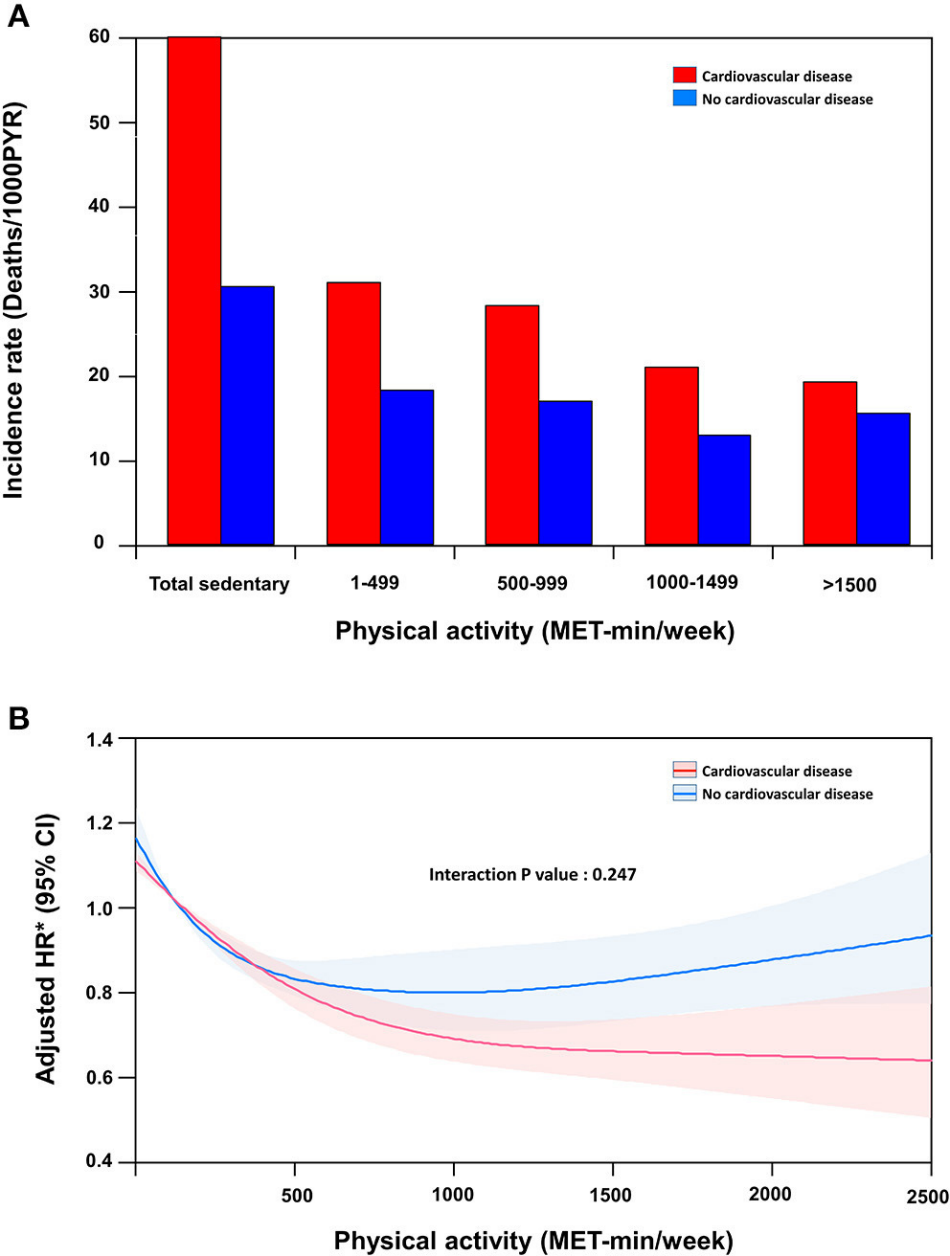
Mortality according to physical activity level stratified by cardiovascular disease. **(A)** Incidence rate (Deaths/1,000 patient years), **(B)** Spline curve for the hazard ratio of a continuous variable.

The median follow-up duration was 42 months (interquartile range, 30–51). Figure 2A shows the incidence rate of mortality per 1,000 person-years stratified according to the presence of CVD and the level of physical activity. In participants without CVD, the unadjusted risk of all-cause mortality showed a J-shaped association with the amount of physical activity. After adjustment for age, sex, body mass index, and other baseline comorbidities, mortality risk was the highest in participants with a sedentary lifestyle and the lowest in participants with a physical activity level of 1000–1499 MET-min/week. A very high level of physical activity (≥1,500 MET-min/week) was associated with a significantly higher risk of mortality than the trough.

In participants with CVD, the unadjusted risk of mortality was reduced with a higher level of physical activity. After adjusting for age, sex, body mass index, and other baseline comorbidities, mortality risk was the highest in participants with a sedentary lifestyle and the lowest in participants with a physical activity level of >1,500 MET-min/week (Table 2). While individuals without CVD benefited the most with physical activity levels between 1,000 and 1,499 MET-min/week, the benefits in those with CVD progressively increased at levels ≥1,500 MET-min/week. The adjusted mortality risk of individuals with CVD who performed a high level of physical activity (≥1,500 MET-min/week) was shown to be lower than that of their counterparts without CVD.

**Table 2:** Leisure-time physical activity and the risk of all-cause mortality stratified according to the presence of cardiovascular disease.

	**Patients**	**Deaths**	**Deaths/1,000 PYR**	**Unadjusted HR (95% CI)**	***P* value**	**Adjusted HR (95% CI)**	***P* value**
**With cardiovascular disease**
Sedentary	9,760	1,739	60.0	Reference		Reference	
1–499 MET-min/week	5,721	560	30.9	0.51 (0.47–0.56)	<0.001	0.69 (0.63–0.76)	<0.001
500–999 MET-min/week	5,178	463	28.2	0.47 (0.42–0.52)	<0.001	0.71 (0.64–0.79)	<0.001
1,000–1,499 MET-min/week	1,444	99	21.0	0.35 (0.28–0.43)	<0.001	0.59 (0.48–0.72)	<0.001
≥1,500 MET-min/week	1,768	112	19.2	0.32 (0.26–0.38)	<0.001	0.52 (0.43–0.63)	<0.001
**Without cardiovascular disease**
Sedentary	15,494	1,567	30.5	Reference		Reference	
1–499 MET-min/week	10,838	668	18.2	0.60 (0.54–0.65)	<0.001	0.75 (0.69–0.83)	<0.001
500–999 MET-min/week	10,782	610	16.9	0.55 (0.50–0.61)	<0.001	0.73 (0.66–0.80)	<0.001
1,000–1,499 MET-min/week	3,321	146	12.9	0.42 (0.35–0.50)	<0.001	0.62 (0.52–0.74)	<0.001
≥1,500 MET-min/week	3,917	204	15.4	0.50 (0.44–0.58)	<0.001	0.72 (0.62–0.83)	<0.001

* *Adjusted for age, sex, body mass index, hypertension, diabetes mellitus, dyslipidemia, chronic kidney disease, chronic obstructive pulmonary disease, malignancy, smoking, alcohol, osteoporosis, hospital frailty risk score, Charlson comorbidity index score*.

*PYR, person-years; MET, metabolic equivalent task; HR, hazard ratio; CI, confidence interval*.

The cubic spline curve is shown in Figure 2B. Every 500 MET-min/week increase in physical activity resulted in an 11% and 16% reduction in the risk of mortality in the non-CVD and CVD groups, respectively (interaction *P* = 0.247). As physical activity increased, the difference in the adjusted hazard ratios between the two groups increased.

The risks of cardiovascular and non-cardiovascular deaths according to the level of physical activity were similar to those of all-cause mortality (Supplementary Tables 5, 6). In the subgroup analysis, the associations between physical activity level and mortality were consistent, regardless of age, sex, body mass index, and other comorbidities (Supplementary Table 7).

### Impact of Physical Activity on All-Cause Mortality According to Specific CVD

The incidence rates and adjusted hazard ratios according to the physical activity level for each CVD are presented in Table 3. The incidence and risk of all-cause death were reduced in the physically active group, irrespective of the specific CVD.

**Table 3:** Leisure-time physical activity and the risk of all-cause mortality stratified according to the presence of each cardiovascular disease.

	**Patients**	**Deaths**	**Deaths/1,000 PYR**	**Adjusted HR (95% CI)**	***P* value**	**Patients**	**Deaths**	**Deaths, /1,000 PYR**	**Adjusted HR (95% CI)**	***P* value**
**Stroke or TIA**	**With previous stroke or TIA**					**Without previous stroke or TIA**				
Sedentary	5,850	1,196	70.61	Reference		19,404	2,110	33.28	Reference	
1–499 MET-min/week	3,178	312	30.90	0.62 (0.54–0.70)	<0.001	13,381	916	20.49	0.77 (0.71–0.83)	<0.001
500–999 MET-min/week	2,905	256	27.85	0.61 (0.53–0.70)	<0.001	13,055	817	18.85	0.77 (0.70–0.83)	<0.001
1,000–1,499 MET-min/week	751	50	20.29	0.49 (0.37–0.66)	<0.001	4,014	195	14.32	0.65 (0.56–0.75)	<0.001
≥1,500 MET-min/week	935	64	20.74	0.49 (0.38-0.63)	<0.001	4750	252	15.75	0.69 (0.60-0.78)	<0.001
**Heart failure**	**With previous heart failure**					**Without previous heart failure**				
Sedentary	3,936	776	67.34	Reference		21,318	2,530	36.76	Reference	
1–499 MET-min/week	2,154	283	42.40	0.82 (0.71–0.94)	0.006	14,405	945	19.64	0.70 (0.64–0.75)	<0.001
500–999 MET-min/week	1,825	218	37.92	0.84 (0.71–0.98)	0.024	14,135	855	18.27	0.69 (0.64–0.75)	<0.001
1,000–1,499 MET-min/week	487	38	24.05	0.56 (0.40–0.79)	<0.001	4,278	207	14.27	0.61 (0.53–0.70)	<0.001
≥1,500 MET-min/week	580	44	23.42	0.54 (0.40–0.74)	<0.001	5,105	272	15.80	0.65 (0.57–0.74)	<0.001
**Myocardial infarction**	**With previous myocardial infarction**					**Without previous myocardial infarction**				
Sedentary	1,273	281	78.12	Reference		23,981	3,025	39.42	Reference	
1–499 MET-min/week	727	96	44.07	0.74 (0.58–0.94)	0.016	15,832	1,132	21.51	0.72 (0.67–0.77)	<0.001
500–999 MET-min/week	662	84	40.68	0.73 (0.57–0.94)	0.015	15,298	989	19.60	0.71 (0.66–0.77)	<0.001
1,000–1,499 MET-min/week	187	21	35.09	0.75 (0.48–1.18)	0.215	4,578	224	14.46	0.59 (0.51–0.68)	<0.001
≥1,500 MET-min/week	277	19	21.39	0.43 (0.27–0.68)	<0.001	5,408	297	16.32	0.65 (0.57–0.73)	<0.001
**Peripheral artery disease**	**With previous peripheral artery disease**					**Without previous peripheral artery disease**				
Sedentary	2,452	321	44.02	Reference		22,802	2,985	40.86	Reference	
1–499 MET-min/week	1,635	134	26.20	0.72 (0.59–0.89)	0.002	14,924	1,094	22.02	0.72 (0.67–0.78)	<0.001
500–999 MET-min/week	1,520	97	20.09	0.61 (0.49–0.78)	<0.001	14,440	976	20.46	0.73 (0.67–0.78)	<0.001
1,000–1,499 MET-min/week	467	35	23.28	0.78 (0.54–1.12)	0.173	4,298	210	14.40	0.58 (0.50–0.67)	<0.001
≥1,500 MET-min/week	549	35	19.68	0.61 (0.43–0.87)	0.006	5,136	281	16.23	0.63 (0.56–0.72)	<0.001

* *Adjusted for age, sex, body mass index, hypertension, diabetes mellitus, dyslipidemia, chronic kidney disease, chronic obstructive pulmonary disease, malignancy, smoking, alcohol, osteoporosis, hospital frailty risk score, Charlson comorbidity index score*.

*PYR, person-years; MET, metabolic equivalent task; HR, hazard ratio; CI, confidence interval*.

**Figure 3:**
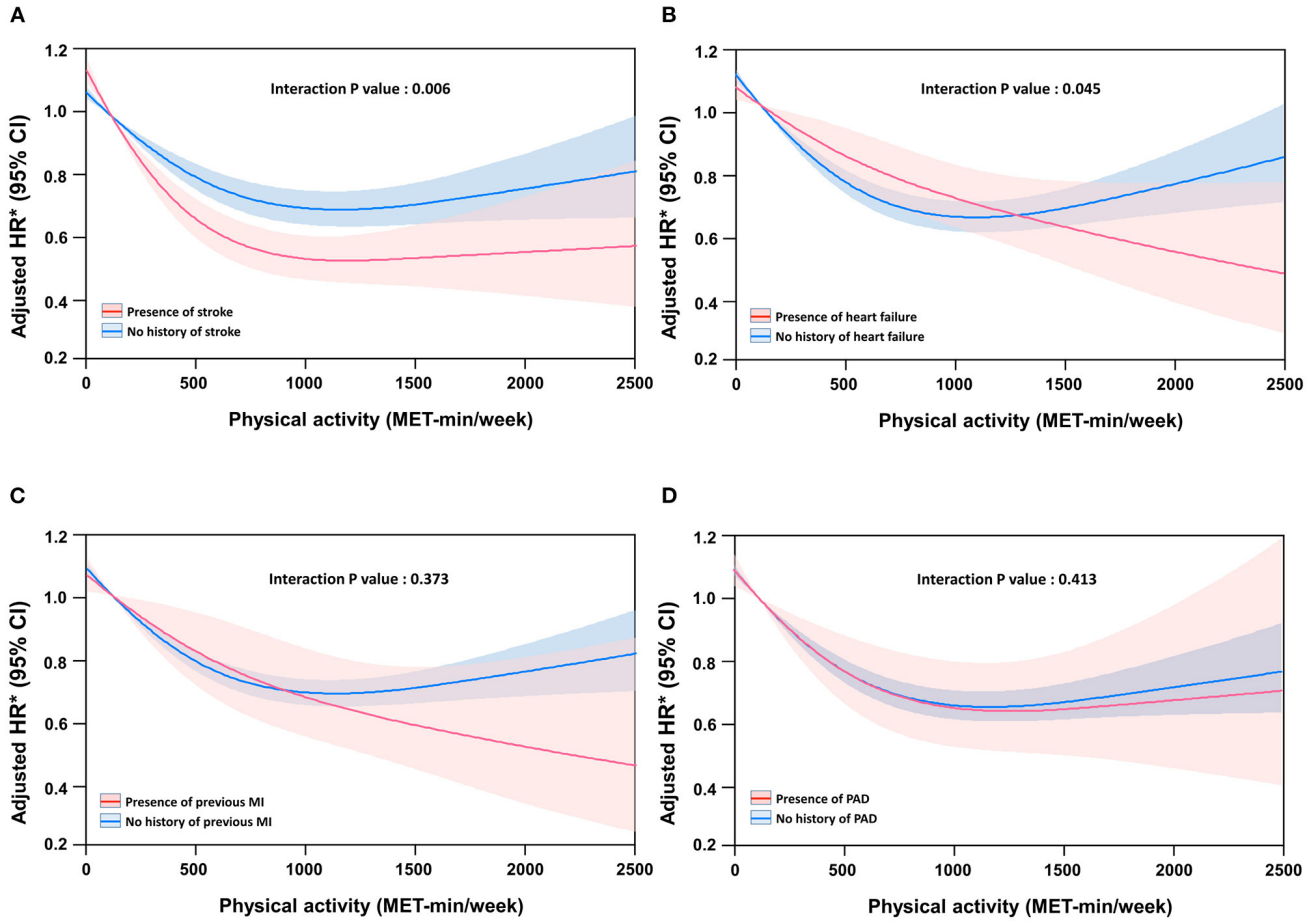
Spline curve of all-cause mortality according to physical activity level in patients with specific cardiovascular disease. **(A)** Stroke, **(B)** heart failure, **(C)** previous myocardial infarction (MI), and **(D)** peripheral artery disease (PAD).

Figure 3 shows the association between the risk of mortality and continuous measures of physical activity using restricted cubic spline curves according to each CVD. The risk of mortality progressively reduced with increase in physical activity in patients with heart failure (Figure 3B) or myocardial infarction (Figure 3C), but reached a plateau in patients with stroke (Figure 3A) or peripheral artery disease (Figure 3D). The differences in reduction in the risk of mortality according to the presence of specific CVD were significant in patients with heart failure and stroke, and showed the same trend in myocardial infarction. A 500 MET-min/week increase in physical activity was associated with a 20% and 11% (interaction *P* = 0.006), and 13 and 11% (interaction *P* = 0.045) reduction in the risk of mortality in participants with and without stroke and with and without heart failure, respectively.

## Discussion

The present study investigated the association between physical activity and mortality according to the presence of specific CVDs in a nationwide elderly population. Our principal finding was that physical activity was associated with a reduced risk of all-cause mortality in older adults with or without CVD, and the benefits of physical activity were greater in patients with CVD than in those without CVD. Second, physical activity was associated with a reduced risk of all-cause mortality in older adults irrespective of the specific CVD. Third, the risk of mortality progressively reduced with increasing physical activity in patients with heart failure or myocardial infarction, but reached a plateau in patients with stroke or peripheral artery disease. Finally, the benefits of physical activity were greater, in patients with stroke or heart failure.

### CVD and Physical Activity

Contemporary guidelines emphasize that physical activity can make people feel better, function better, sleep better, and reduce the risk of many chronic diseases. The suggested target range of physical activity is 500–1,000 MET-min/week of aerobic physical activity, which is equivalent to 150–300 min of moderate-intensity or 75–150 min of vigorous physical activity per week. This recommendation is based on observations that the greatest survival benefit is provided by achieving a physical activity level of 500–1,000 MET-min/week (2). The findings of the present study are consistent with these conclusions.

Previous studies that showed the survival benefits of physical activity primarily focused on healthy individuals and did not consider the presence of CVD (or specific CVD) (7–9). The present study provides a novel perspective on the preventive role of physical activity in patients with CVD in an elderly population. In the present study, individuals with CVD tended to have lower level of physical activity. Not only were they typically older with multiple comorbidities, but their cardiac condition also limited their physical capacity. Nevertheless, clinicians should emphasize the importance of a physically active lifestyle for those patients, as they may experience greater benefits than their counterparts without CVD at the same levels of physical activity.

### Specific CVD and Physical Activity

Strong evidence supports exercise-based cardiac rehabilitation in patients with coronary heart disease and exercise training in patients with chronic heart failure (26–28). In previous studies on peripheral artery disease, low-intensity exercise was significantly less effective than high-intensity exercise and was not significantly different from the non-exercise control in improving the 6-min walk distance (29). However, recommendations on the level of physical activity for specific CVD groups are not consistent. The European Society of Cardiology guidelines on CVD prevention encourage at least 150–300 min/week of moderate-intensity or 75–150 min/week of vigorous aerobic physical activity (13). Meanwhile, the guidelines for stable ischemic heart disease from the American College of Cardiology Foundation and American Heart Association recommend 30–60 min of moderate-intensity aerobic activity for at least 5 days and preferably 7 days per week (30). The latter states that the recommended level of physical activity is in line with that recommended for healthy adults (31, 32).

In this study, physical activity was associated with a reduced risk of all-cause mortality in older adults irrespective of the specific CVD, but the effect was greater in patients with stroke or heart failure. Notably, the risk of mortality was progressively reduced by increasing physical activity in patients with heart failure or myocardial infarction. The benefits were not significantly different between patients with and without peripheral artery disease. In the case of peripheral artery disease, there is a possibility that the effect of physical activity was not fully elucidated, as peripheral artery disease may have restricted the amount of exercise. However, all data suggest that a physical activity level of 500 MET-min/week should be considered as the minimum requirement for patients with CVD.

### Limitations

This study had several limitations. First, this retrospective and non-randomized cohort study cannot prove or disprove causal relationships. Second, we relied on self-reported questionnaires of physical activity collected at specific time points. The questionnaires surveyed lifestyle behaviors during the preceding 1 week. Information obtained from questionnaires may not represent the level of actual physical activity, and behavioral changes that occur during follow-up could be assessed in our study. However, the large sample size of this study reduced the level of potential uncertainty and provided a reliable approximation of the level of physical activity at the population level. Third, only physical activity during leisure time was analyzed, but various types of physical activity, such as household, occupation, and transportation, that can occur throughout the day were not included or analyzed. Firth, the existence of unadjusted confounders could not be excluded despite strict statistical adjustments. Confounders such as diet habits, changes in medication use, and compliance were not adjusted for in the analysis. Finally, the existence of CVD was determined using claims data, so there is a possibility of errors due to incorrect coding. To minimize this problem, definitions that were verified in previous studies using sample cohorts of the Korean NHIS were applied, and the diagnostic reliability of the cohort data was high (15–24).

## Conclusion

Physical activity is important for improved outcomes in elderly patients with CVD and should be routinely recommended. Physical activity was associated with a reduced risk of all-cause mortality in older adults with or without CVD. The benefits of physical activity were greater in patients with CVD, especially patients with stroke of heart failure, than those in patients without CVD.

## Data Availability Statement

The original contributions presented in the study are included in the article/Supplementary Material, further inquiries can be directed to the corresponding authors.

## Ethics Statement

The studies involving human participants were reviewed and approved by the Institutional Review Board of the Yonsei University Health System (4-2021-0894). Written informed consent for participation was not required for this study in accordance with the national legislation and the institutional requirements.

## Author Contributions

M-HK and J-HS contributed to the conception and design of the work, interpretation of data, and drafting of the manuscript. P-SY and BJ are joint senior authors and contributed to the conception and design of the work and critical revision of the manuscript. EJ and P-SY contributed to the acquisition and analysis of data. M-NJ, HY, T-HK, H-NP, M-HL, and GL contributed to the conception and design of the work and revision of the manuscript. All authors read and approved the manuscript before its submission.

## Funding

This research was supported by a grant of Patient-Centered Clinical Research Coordinating Center (PACEN) funded by the Ministry of Health and Welfare, Republic of Korea (grant numbers: HI19C0481, HC19C013, and HI15C1200).

## Conflict of Interest

GL has served as a consultant and speaker for BMS/Pfizer, Boehringer Ingelheim, and Daiichi-Sankyo. No fees were received either directly or personally. BJ has served as a speaker for Bayer, BMS/Pfizer, Medtronic, and Daiichi-Sankyo and received research funds from Medtronic and Abbott. No fees were received either directly or personally. The remaining authors declare that the research was conducted in the absence of any commercial or financial relationships that could be construed as a potential conflict of interest.

## Publisher's Note

All claims expressed in this article are solely those of the authors and do not necessarily represent those of their affiliated organizations, or those of the publisher, the editors and the reviewers. Any product that may be evaluated in this article, or claim that may be made by its manufacturer, is not guaranteed or endorsed by the publisher.
