# Supplementary material for: Impact of Physical Activity on All-Cause Mortality According to Specific Cardiovascular Disease
Source: Front Cardiovasc Med. 2022 Feb 4;9:811058. doi: 10.3389/fcvm.2022.811058 (PMC8855984; doi:10.3389/fcvm.2022.811058)
Supplement: Supplementary file 1 [file Data_Sheet_1.docx]

**
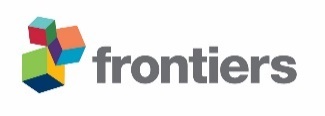
**

Supplementary Material

**Supplementary Table 1.** Definition of cardiovascular disease

|  | **Definitions** | **ICD-10 codes or conditions** |
| --- | --- | --- |
| **Comorbidities** |  |  |
| Atrial fibrillation[^1^](#_ENREF_1)^-3^ | Defined from diagnosis* | ICD-10: I48 |
| Heart failure[^2^](#_ENREF_2)^,3^ | Defined from diagnosis* | ICD-10: I11.0, I50, I97.1 |
| Hypertension[^2^](#_ENREF_2)^,3,4^ | Defined from diagnosis* | ICD-10: I10, I11, I12, I13, I15 and antihypertensive medication |
| Diabetes mellitus[^2^](#_ENREF_2)^,3^ | Defined from diagnosis* plus treatment | ICD-10: E10, E11, E12, E13, E14  Treatment: all kinds of oral antidiabetics and insulin. |
| Dyslipidemia[^2^](#_ENREF_2)^,3^ | Defined from diagnosis* | ICD-10: E78 |
| Ischemic stroke[^2^](#_ENREF_2)^,3^ | Defined from diagnosis* | ICD-10: I63, I64 |
| Transient ischemic attack[^2^](#_ENREF_2)^,3^ | Defined from diagnosis* | ICD-10: G45 |
| Hemorrhagic stroke | Defined from diagnosis* | ICD-10: I60, I61, I62 |
| Myocardial infarction[^5^](#_ENREF_3) | Defined from diagnosis* | ICD-10: I21, I22, I25.2 |
| Coronary heart disease | Defined from a history of acute myocardial infarction, coronary revascularization, or chronic ischemic heart disease. | Acute myocardial infarction: admission diagnosis (ICD-10: I21, I22) concurrently with coronary angiography (HA670, HA680, HA681)  Coronary revascularization: percutaneous coronary intervention (M6551, M6552, M6561, M6563, M6562, M6564, M6571, M6572), thrombolytic treatment (M6634), or coronary artery bypass graft (O1641, OA641, O1642, OA642, O1647, OA647)  Chronic ischemic heart disease: diagnosis* (ICD-10: I25.2, I25.5, I25.6, I25.8, I25.9) |
| Peripheral arterial disease^2,3^ | Defined from diagnosis* | ICD-10: I70.0, I70.1, I70.2, I70.8, I70.9 |
| Chronic kidney disease^2,3^ | Defined from eGFR or diagnosis*  (if laboratory value was not available, diagnosis code was used) | eGFR <60mL/min per 1.73 m^2^  ICD-10: N18, N19 |
| End-stage renal disease^6^ | Defined from national registry for severe illness. | Patients with end-stage renal disease undergoing chronic dialysis or received a kidney transplant. |
| Hypertrophic cardiomyopathy^7^ | Defined from at least one records of either inpatient or outpatient diagnoses | ICD-10: I42.1, I42.2 |
| Sleep apnea | Defined from diagnosis* | ICD-10: G47.3 |
| Proteinuria | Defined from laboratory data (if laboratory value was not available, diagnosis code was used) | Urine dipstick proteinuria 1+ or higher (ICD-10: N06, N391, N392, R80) |
| Osteoporosis^8^ | Defined from diagnosis* | ICD-10: M80, M81, M82 (except M82.0) |
| Hyperthyroidism | Defined from diagnosis* | ICD-10: E05 |
| Hypothyroidism | Defined from diagnosis* | ICD-10: E03 |
| Chronic Liver disease | Defined from diagnosis of chronic liver disease, cirrhosis, and hepatitis | ICD-10: B18, K70, K71, K72, K73, K74, K76.1 |
| Chronic obstructive pulmonary disease^9^ | Defined from diagnosis* plus treatment | ICD-10: J42, J43(except J43.0), J44  Treatment: SABA, SAMA, LABA, LAMA, ICS, ICS+LABA, or methylxanthine (>1 months). |
| Malignancy | Defined from diagnoses of cancer (non-benign) | ICD-10: C00-C97 |
| **Clinical outcomes** |  |  |
| Coronary heart disease | Defined from an event of acute myocardial infarction, coronary revascularization, or death of which the cause was recorded as a coronary artery disease or myocardial infarction | Acute myocardial infarction: admission diagnosis (ICD-10: I21, I22) concurrently with coronary angiography (HA670, HA680, HA681)  Coronary revascularization: percutaneous coronary intervention (M6551, M6552, M6561, M6563, M6562, M6564, M6571, M6572), thrombolytic treatment (M6634), or coronary artery bypass graft (O1641, OA641, O1642, OA642, O1647, OA647)  Coronary artery disease or myocardial infarction:  ICD-10: I20, I21, I22, I23, I25 |
| Ischemic stroke^1,3^ | Defined from any discharge diagnoses with concomitant imaging studies | ICD-10: I63, I64 |
| Systemic embolism | Defined from admission diagnosis or related death | ICD-10: I74, N280 (including renal infarction) |

Abbreviations: eGFR, estimated glomerular filtration rate; ICD-10, International Classification of Diseases-10th Revision.

*To ensure accuracy, comorbidities were established based on one inpatient or two outpatient records of ICD-10 codes in the database.

**References for supplementary Table 1.**

1. Kim D, Yang PS, Jang E, et al. Increasing trends in hospital care burden of atrial fibrillation in Korea, 2006 through 2015. Heart 2018.
2. Lee H, Kim TH, Baek YS, et al. The Trends of Atrial Fibrillation-Related Hospital Visit and Cost, Treatment Pattern and Mortality in Korea: 10-Year Nationwide Sample Cohort Data. Korean Circ J 2017;47:56-64.
3. Kim TH, Yang PS, Kim D, et al. CHA2DS2-VASc Score for Identifying Truly Low-Risk Atrial Fibrillation for Stroke: A Korean Nationwide Cohort Study. Stroke 2017;48:2984-2990.
4. Kim D, Yang PS, Kim TH, et al. Ideal blood pressure in patients with atrial fibrillation. J Am Coll Cardiol. 2018;72:1233-1245.
5. Lee HY, Yang PS, Kim TH, et al. Atrial fibrillation and the risk of myocardial infarction: a nation-wide propensity-matched study. Sci Rep 2017;7:12716.
6. Seong SC, Kim YY, Park SK, et al. Cohort profile: the National Health Insurance Service-National Health Screening Cohort (NHIS-HEALS) in Korea. BMJ Open 2017;7:e016640.
7. Jung H, Yang PS, Jang E, et al. Effectiveness and Safety of Non-Vitamin K Antagonist Oral Anticoagulants in Atrial Fibrillation Patients with Hypertrophic Cardiomyopathy: A Nationwide Cohort Study. CHEST. 201;155:354-363.
8. Kim D, Yang PS, Kim TH,et al. Effect of Atrial Fibrillation on the Incidence and Outcome of Osteoporotic Fracture - A Nationwide Population-Based Study. Circ J. 2018 25;82:1999-2006.
9. Song S, Yang PS, Kim TH, et al. Relation of Chronic Obstructive Pulmonary Disease to Cardiovascular Disease in the General Population. Am J Cardiol 2017;120:1399-1404.

**Supplementary Table 2.** Baseline characteristics according to leisure-time physical activity

| **Variables** | **Sedentary**  **(N=25254)** | **1-499 MET-min/week**  **(N=16559)** | **500-999 MET-min/week**  **(N=15960)** | **1000-1499 MET-min/week**  **(N=4765)** | **≥1500 MET-min/week**  **(N=5685)** | **P value** |
| --- | --- | --- | --- | --- | --- | --- |
| **Leisure time physical activity** | 0.0 ± 0.0 | 301.3 ± 129.9 | 711.5 ± 101.2 | 1187.8 ± 120.7 | 2035.4 ± 445.4 | <0.001 |
| **Demographic** |  |  |  |  |  |  |
| Age, years | 74.9 ± 6.4 | 73.4 ± 5.5 | 72.8 ± 4.9 | 71.9 ± 4.4 | 72.0 ± 4.4 | <0.001 |
| Male | 8185 (32.4) | 5700 (34.4) | 6809 (42.7) | 2489 (52.2) | 3300 (58.0) | <0.001 |
| Body mass index | 23.5 ± 3.6 | 23.9 ± 3.4 | 23.8 ± 3.2 | 23.9 ± 3.1 | 24.0 ± 3.1 | <0.001 |
| Waist | 82.5 ± 9.4 | 83.2 ± 8.8 | 83.3 ± 8.6 | 83.6 ± 8.5 | 83.9 ±8.4 | <0.001 |
| Systolic blood pressure | 131.2 ± 17.5 | 131.3 ± 16.8 | 131.3 ± 16.9 | 131.0 ± 16.5 | 131.9 ± 16.6 | 0.034 |
| Diastolic blood pressure | 78.3 ± 10.7 | 78.3 ± 10.4 | 78.1 ± 10.2 | 77.9 ±10.0 | 78.4 ± 10.4 | 0.063 |
| Smoking | 4869 (19.3) | 3734 (22.5) | 4592 (28.8) | 1568 (32.9) | 1955 (34.4) | <0.001 |
| Alcohol | 3563 (14.1) | 3118 (18.8) | 3597 (22.5) | 1452 (30.5) | 1701 (29.9) | <0.001 |
| **Income status** |  |  |  |  |  | <0.001 |
| Low | 8279 (32.8) | 4948 (29.9) | 4817 (30.2) | 1306 (27.4) | 1577 (27.7) |  |
| Mid | 5694 (22.5) | 3634 (21.9) | 3467 (21.7) | 1014 (21.3) | 1209 (21.3) |  |
| High | 11281 (44.7 | 7977 (48.2) | 7676(48.1) | 2445 (51.3) | 2899 (51.0) |  |
| **Risk scores** |  |  |  |  |  |  |
| Hospitality frailty risk score | 2.8 ± 5.4 | 1.7 ± 3.4 | 1.4 ± 3.0 | 1.2 ± 2.7 | 1.2 ± 2.8 | <0.001 |
| Charlson comorbidity index | 3.3 ± 2.8 | 3.2 ± 2.7 | 3.1 ± 2.7 | 3.0 ± 2.6 | 3.0 ± 2.7 | <0.001 |
| **Comorbidities** |  |  |  |  |  |  |
| Hypertension | 15835 (62.7) | 10187 (61.5) | 9508 (59.6) | 2804 (58.8) | 3392 (59.7) | <0.001 |
| Diabetes mellitus | 5293 (21.0) | 3533 (21.3) | 3539 (22.2) | 1080 (22.7) | 1322 (23.3) | <0.001 |
| Dyslipidemia | 12458 (49.3) | 8699 (52.5) | 8348 (52.3) | 2570 (53.9) | 2973 (52.3) | <0.001 |
| Chronic kidney disease | 820 (3.2) | 458 (2.8) | 407 (2.6) | 118 (2.5) | 134 (2.4) | <0.001 |
| Heart failure | 3936 (15.6) | 2154 (13.0) | 1825 (11.4) | 487 (10.2) | 580 (10.2) | <0.001 |
| Vascular disease | 3440 (13.6) | 2202 (13.3) | 2050 (12.8) | 617 (12.9) | 771 (13.6) | 0.201 |
| Ischemic stroke or TIA | 5660 (22.4) | 3083 (18.6) | 2799 (17.5) | 730 (15.3) | 900 (15.8) | <0.001 |
| COPD | 3539 (14.0) | 2040 (12.3) | 1857 (11.6) | 486 (10.2) | 583 (10.3) | <0.001 |
| Malignancy | 4002 (15.8) | 2740 (16.5) | 2597 (16.3) | 791 (16.6) | 1011 (17.8) | 0.007 |
| Osteoporosis | 11693 (46.3) | 7288 (44.0) | 6103 (38.2) | 1636 (34.3) | 1756 (30.9) | <0.001 |
| Previous MI | 1273 (5.0) | 727 (4.4) | 662 (4.1) | 187 (3.9) | 277 (4.9) | <0.001 |
| Peripheral artery disease | 2452 (9.7) | 1635 (9.9) | 1520 (9.5) | 467 (9.8) | 549 (9.7) | 0.878 |
| **Laboratory findings** |  |  |  |  |  |  |
| Fasting blood glucose | 105.7 ± 32.7 | 105.7 ± 30.9 | 106.2 ± 30.5 | 106.7 ± 30.5 | 107.6 ± 32.4 | 0.001 |
| Total cholesterol | 196.2 ± 41.5 | 197.4 ± 40.4 | 195.5 ± 39.7 | 195.0 ± 39.5 | 194.2 ± 39.1 | <0.001 |
| Triglyceride | 141.2 ± 80.5 | 141.2 ± 80.2 | 139.0 ± 82.6 | 139.1 ± 81.8 | 135.1 ± 79.2 | <0.001 |
| LDL-cholesterol | 116.8 ± 39.9 | 116.7 ± 37.4 | 115.2 ± 36.5 | 114.3 ± 35.9 | 114.2 ± 36.0 | <0.001 |
| HDL-cholesterol | 53.0 ± 30.7 | 52.9 ± 21.2 | 53.1 ± 22.1 | 53.2 ± 20.2 | 53.5 ± 23.7 | 0.604 |
| AST | 26.4 ± 20.0 | 26.4 ± 19.8 | 26.8 ± 19.8 | 26.9 ± 15.0 | 26.7 ± 13.3 | 0.096 |
| ALT | 21.1 ± 17.8 | 21.9 ± 17.2 | 22.5 ± 20.5 | 23.0 ± 17.4 | 22.8 ± 14.1 | <0.001 |
| Gamma-GT | 32.6 ± 52.3 | 32.8 ± 49.5 | 34.0 ± 52.9 | 36.1 ± 55.3 | 37.5 ± 60.6 | <0.001 |
| Serum creatinine | 1.0 ± 1.0 | 1.0 ± 0.9 | 1.0 ± 0.9 | 1.0 ± 1.0 | 1.0 ± 1.6 | 0.004 |
| eGFR | 70.0 ± 18.3 | 71.3 ± 17.7 | 72.0 ± 17.4 | 72.9 ± 17.3 | 72.5 ± 16.8 | <0.001 |

* Values are presented as mean ± standard deviation, median (Q1, Q3, quartiles [25th and 75th percentiles]), or %.

COPD, chronic obstructive pulmonary disease; TIA, transient ischemic attack; MI, myocardial infarction; LDL, low-density lipoprotein; HDL, high-density lipoprotein; AST, aspartate aminotransferase; ALT, alanine aminotransferase; Gamma-GT, gamma-glutamyl transpeptidase; eGFR, estimated glomerular filtration rate.

**Supplementary Table 3.** Baseline characteristics of participants with cardiovascular disease according to leisure-time physical activity

| **Variables** | **Sedentary**  **(N=9760)** | **1-499 MET-min/week**  **(N=5721)** | **500-999 MET-min/week**  **(N=5178)** | **1000-1499 MET-min/week**  **(N=1444)** | **≥1500 MET-min/week**  **(N=1768)** | **P value** |
| --- | --- | --- | --- | --- | --- | --- |
| **Leisure time physical activity** | 0.0 ± 0.0 | 296.2 ± 130.1 | 710.7 ± 100.0 | 1180.0 ± 117.6 | 2033.5 ± 447.3 | <0.001 |
| **Demographic** |  |  |  |  |  |  |
| Age, years | 76.1 ± 6.5 | 74.4 ± 5.7 | 73.6 ± 5.2 | 72.5 ± 4.7 | 72.6 ± 4.6 | <0.001 |
| Male | 2782 (28.5) | 1792 (31.3) | 2058 (39.7) | 680 (47.1) | 987 (55.8) | <0.001 |
| Body mass index | 23.7 ± 3.8 | 24.3 ± 3.5 | 24.2 ± 3.3 | 24.4 ± 3.1 | 24.4 ± 3.0 | <0.001 |
| Waist | 83.2 ± 9.9 | 84.4 ± 9.0 | 84.4 ± 8.5 | 84.8 ± 8.5 | 84.9 ± 8.4 | <0.001 |
| Systolic blood pressure | 130.8 ± 17.8 | 131.2 ± 16.8 | 131.9 ± 16.9 | 131.6 ± 16.3 | 131.7 ± 16.6 | 0.003 |
| Diastolic blood pressure | 77.9 ± 10.8 | 78.0 ± 10.6 | 78.1 ± 10.3 | 78.0 ± 9.9 | 77.6 ± 10.3 | 0.607 |
| Smoking | 1581 (16.2) | 1181 (20.6) | 1369 (26.4) | 442 (30.6) | 563 (31.8) | <0.001 |
| Alcohol | 911 (9.3) | 822 (14.4) | 915 (17.7) | 340 (23.5) | 442 (25.0) | <0.001 |
| **Income status** |  |  |  |  |  | <0.001 |
| Low | 3189 (32.7%) | 1646 (28.8%) | 1552 (30.0%) | 378 (26.2%) | 449 (25.4%) |  |
| Mid | 2109 (21.6%) | 1227 (21.4%) | 1075 (20.8%) | 316 (21.9%) | 383 (21.7%) |  |
| High | 4462 (45.7%) | 2848 (49.8%) | 2551 (49.3%) | 750 (51.9%) | 936 (52.9%) |  |
| **Risk scores** |  |  |  |  |  |  |
| Hospitality frailty risk score | 4.9 ± 7.2 | 3.1 ± 4.8 | 2.7 ± 4.3 | 2.2 ± 3.7 | 2.3 ± 4.1 | <0.001 |
| Charlson comorbidity index | 5.1 ± 2.8 | 5.0 ± 2.8 | 4.8 ± 2.7 | 4.7 ± 2.6 | 4.8 ± 2.7 | <0.001 |
| **Comorbidities** |  |  |  |  |  |  |
| Hypertension | 8370 (85.8) | 4839 (84.6) | 4352 (84.0) | 1205 (83.4) | 1513 (85.6) | 0.018 |
| Diabetes mellitus | 2916 (29.9) | 1806 (31.6) | 1655 (32.0) | 474 (32.8) | 579 (32.7) | 0.009 |
| Dyslipidemia | 6669 (68.3) | 4181 (73.1) | 3884 (75.0) | 1118 (77.4) | 1350 (76.4) | <0.001 |
| Chronic kidney disease | 552 (5.7) | 285 (5.0) | 247 (4.8) | 66 (4.6) | 86 (4.9) | 0.085 |
| Heart failure | 3936 (40.3) | 2154 (37.7) | 1825 (35.2) | 487 (33.7) | 580 (32.8) | <0.001 |
| Vascular disease | 3440 (35.2) | 2202 (38.5) | 2050 (39.6) | 617 (42.7) | 771 (43.6) | <0.001 |
| Ischemic stroke or TIA | 5660 (58.0) | 3083 (53.9) | 2799 (54.1) | 730 (50.6) | 900 (50.9) | <0.001 |
| COPD | 1941 (19.9) | 1056 (18.5) | 820 (15.8) | 216 (15.0) | 269 (15.2) | <0.001 |
| Malignancy | 1948 (20.0) | 1194 (20.9) | 1040 (20.1) | 291 (20.2) | 408 (23.1) | 0.039 |
| Osteoporosis | 5429 (55.6) | 2997 (52.4) | 2402 (46.4) | 638 (44.2) | 684 (38.7) | <0.001 |
| Previous MI | 1273 (13.0) | 727 (12.7) | 662 (12.8) | 187 (13.0) | 277 (15.7) | 0.021 |
| Peripheral artery disease | 2452 (25.1) | 1635 (28.6) | 1520 (29.4) | 467 (32.3) | 549 (31.1) | <0.001 |
| **Laboratory findings** |  |  |  |  |  |  |
| Fasting blood glucose | 108.0 ± 35.6 | 108.5 ± 34.2 | 108.4 ± 33.0 | 109.8 ± 35.2 | 109.9 ± 33.3 | 0.146 |
| Total cholesterol | 191.3 ± 42.6 | 191.2 ± 41.6 | 190.1 ± 40.8 | 189.6 ± 42.2 | 187.8 ± 39.7 | 0.008 |
| Triglyceride | 143.2 ± 79.5 | 143.7 ± 81.5 | 141.1 ± 81.4 | 140.6 ± 83.5 | 138.8 ± 82.2 | 0.092 |
| LDL-cholesterol | 113.0 ± 41.1 | 111.2 ± 37.2 | 110.4 ± 36.3 | 109.9 ± 37.6 | 108.1 ± 35.3 | <0.001 |
| HDL-cholesterol | 51.1 ± 27.2 | 51.9 ± 21.3 | 51.7 ± 20.6 | 51.5 ± 13.6 | 52.4 ± 22.4 | 0.127 |
| AST | 25.8 ± 20.5 | 25.9 ± 17.9 | 26.4 ± 17.7 | 26.7 ± 15.0 | 26.7 ± 13.7 | 0.103 |
| ALT | 20.6 ± 16.5 | 21.7 ± 13.5 | 22.5 ± 22.2 | 23.1 ± 18.2 | 23.3 ± 15.0 | <0.001 |
| Gamma-GT | 31.5 ± 44.6 | 32.8 ± 46.2 | 33.2 ± 47.1 | 34.9 ± 42.3 | 38.3 ± 58.3 | <0.001 |
| Serum creatinine | 1.1 ± 1.0 | 1.0 ± 1.0 | 1.0 ± 0.9 | 1.1 ± 1.5 | 1.2 ± 2.6 | 0.009 |
| eGFR | 66.6 ± 19.5 | 67.7 ± 18.9 | 68.8 ± 18.6 | 70.5 ± 18.7 | 69.8 ± 18.7 | <0.001 |

* Values are presented as mean ± standard deviation, median (Q1, Q3, quartiles [25th and 75th percentiles]), or %.

COPD, chronic obstructive pulmonary disease; TIA, transient ischemic attack; MI, myocardial infarction; LDL, low-density lipoprotein; HDL, high-density lipoprotein; AST, aspartate aminotransferase; ALT, alanine aminotransferase; Gamma-GT, gamma-glutamyl transpeptidase; eGFR, estimated glomerular filtration rate.

**Supplementary Table 4.** Baseline characteristics of participants without cardiovascular disease according to leisure-time physical activity

| **Variables** | **Sedentary**  **(N=15494)** | **1-499 MET-min/week**  **(N=10838)** | **500-999 MET-min/week**  **(N=10782)** | **1000-1499 MET-min/week**  **(N=3321)** | **≥1500 MET-min/week**  **(N=3917)** | **P value** |  |
| --- | --- | --- | --- | --- | --- | --- | --- |
| **Leisure time physical activity** | 0.0 ± 0.0 | 303.9 ± 129.8 | 712.0 ± 101.8 | 1191.2 ± 121.9 | 2036.2 ± 444.5 | <0.001 |  |
| **Demographic** |  |  |  |  |  |  |  |
| Age, years | 74.1 ± 6.2 | 72.9 ± 5.3 | 72.5 ± 4.7 | 71.6 ± 4.2 | 71.8 ± 4.3 | <0.001 |  |
| Male | 5403 (34.9) | 3908 (36.1) | 4751 (44.1) | 1809 (54.5) | 2313 (59.1) | <0.001 |  |
| Body mass index | 23.4 ± 3.5 | 23.7 ± 3.3 | 23.6 ± 3.2 | 23.7 ± 3.0 | 23.8 ± 3.1 | <0.001 |  |
| Waist | 82.1 ± 9.1 | 82.6 ± 8.7 | 82.7 ± 8.5 | 83.1 ± 8.4 | 83.4 ± 8.4 | <0.001 |  |
| Systolic blood pressure | 131.5 ± 17.4 | 131.4 ± 16.8 | 131.1 ± 16.9 | 130.7 ± 16.5 | 132.0 ± 16.6 | 0.004 |  |
| Diastolic blood pressure | 78.5 ± 10.6 | 78.5 ± 10.3 | 78.2 ± 10.2 | 77.9 ± 10.1 | 78.7 ± 10.5 | <0.001 |  |
| Smoking | 3288 (21.2) | 2553 (23.6) | 3223 (29.9) | 1126 (33.9) | 1392 (35.5) | <0.001 |  |
| Alcohol | 2652 (17.1) | 2296 (21.2) | 2682 (24.9) | 1112 (33.5) | 1259 (32.1) | <0.001 |  |
| **Income status** |  |  |  |  |  | <0.001 | |
| Low | 5090 (32.9%) | 3302 (30.5%) | 3265 (30.3%) | 928 (27.9%) | 1128 (28.8%) |  | |
| Mid | 3585 (23.1%) | 2407 (22.2%) | 2392 (22.2%) | 698 (21.0%) | 826 (21.1%) |  |  |
| High | 6819 (44.0%) | 5129 (47.3%) | 5125 (47.5%) | 1695 (51.0%) | 1963 (50.1%) |  |  |
| **Risk scores** |  |  |  |  |  |  |  |
| Hospitality frailty risk score | 1.4 ± 3.1 | 0.9 ± 2.1 | 0.8 ± 2.0 | 0.8 ± 1.9 | 0.8 ± 1.8 | <0.001 |  |
| Charlson comorbidity index | 2.2 ± 2.1 | 2.2 ± 2.1 | 2.2 ± 2.2 | 2.2 ± 2.2 | 2.2 ± 2.2 | 0.873 |  |
| **Comorbidities** |  |  |  |  |  |  |  |
| Hypertension | 7465 (48.2) | 5348 (49.3) | 5156 (47.8) | 1599 (48.1) | 1879 (48.0) | 0.202 |  |
| Diabetes mellitus | 2377 (15.3) | 1727 (15.9) | 1884 (17.5) | 606 (18.2) | 743 (19.0) | <0.001 |  |
| Dyslipidemia | 5789 (37.4) | 4518 (41.7) | 4464 (41.4) | 1452 (43.7) | 1623 (41.4) | <0.001 |  |
| Chronic kidney disease | 268 (1.7) | 173 (1.6) | 160 (1.5) | 52 (1.6) | 48 (1.2) | 0.195 |  |
| COPD | 1598 (10.3) | 984 (9.1) | 1037 (9.6) | 270 (8.1) | 314 (8.0) | <0.001 |  |
| Malignancy | 2054 (13.3) | 1546 (14.3) | 1557 (14.4) | 500 (15.1) | 603 (15.4) | 0.001 |  |
| Osteoporosis | 6264 (40.4) | 4291 (39.6) | 3701 (34.3) | 998 (30.1) | 1072 (27.4) | <0.001 |  |
| **Laboratory findings** |  |  |  |  |  |  |  |
| Fasting blood glucose | 104.3 ± 30.6 | 104.3 ± 29.0 | 105.2 ± 29.2 | 105.3 ± 28.1 | 106.6 ± 31.9 | <0.001 |  |
| Total cholesterol | 199.3 ± 40.5 | 200.6 ± 39.4 | 198.2 ± 38.8 | 197.3 ± 38.0 | 197.0 ± 38.6 | <0.001 |  |
| Triglyceride | 139.9 ± 81.1 | 139.9 ± 79.4 | 138.0 ± 83.2 | 138.5 ± 81.1 | 133.4 ± 77.7 | <0.001 |  |
| LDL-cholesterol | 119.3 ± 38.9 | 119.7 ± 37.2 | 117.5 ± 36.4 | 116.2 ± 35.0 | 117.0 ± 35.9 | <0.001 |  |
| HDL-cholesterol | 54.2 ± 32.7 | 53.5 ± 21.1 | 53.8 ± 22.7 | 53.9 ± 22.5 | 54.0 ± 24.2 | 0.218 |  |
| AST | 26.7 ± 19.7 | 26.6 ± 20.7 | 26.9 ± 20.7 | 27.0 ± 15.0 | 26.8 ± 13.1 | 0.681 |  |
| ALT | 21.5 ± 18.6 | 22.1 ± 18.8 | 22.5 ± 19.6 | 23.0 ± 17.0 | 22.6 ± 13.7 | <0.001 |  |
| Gamma-GT | 33.3 ± 56.6 | 32.7 ± 51.2 | 34.5 ± 55.5 | 36.5 ± 60.0 | 37.1 ± 61.6 | <0.001 |  |
| Serum creatinine | 1.0 ± 0.9 | 0.9 ± 0.8 | 1.0 ± 0.8 | 1.0 ± 0.7 | 1.0 ± 0.7 | 0.048 |  |
| eGFR | 72.2 ± 17.2 | 73.2 ± 16.7 | 73.6 ± 16.6 | 73.9 ± 16.6 | 73.7 ± 15.7 | <0.001 |  |

* Values are presented as mean ± standard deviation, median (Q1, Q3, quartiles [25th and 75th percentiles]), or number (%).

COPD, chronic obstructive pulmonary disease; TIA, transient ischemic attack; MI, myocardial infarction; LDL, low-density lipoprotein; HDL, high-density lipoprotein; AST, aspartate aminotransferase; ALT, alanine aminotransferase; Gamma-GT, gamma-glutamyl transpeptidase; eGFR, estimated glomerular filtration rate.

**Supplementary Table 5.** Leisure-time physical activity and risk of cardiovascular death stratified according to the presence of cardiovascular disease

|  | **Patients** | **Deaths** | **Deaths, /1000PYR** | **Unadjusted HR (95% CI)** | **P value** | **Adjusted HR (95% CI)** | **P value** |
| --- | --- | --- | --- | --- | --- | --- | --- |
| **With cardiovascular disease** | | | | | | | |
| Sedentary | 9760 | 578 | 19.65 | Reference |  | Reference |  |
| 1-499 MET-min/week | 5721 | 167 | 9.09 | 0.46 (0.39-0.55) | <0.001 | 0.68 (0.57-0.81) | <0.001 |
| 500-999 MET-min/week | 5178 | 132 | 7.92 | 0.40 (0.33-0.48) | <0.001 | 0.66 (0.54-0.81) | <0.001 |
| 1000-1499 MET-min/week | 1444 | 40 | 8.37 | 0.42 (0.31-0.58) | <0.001 | 0.78 (0.57-1.09) | 0.145 |
| ≥1500 MET-min/week | 1768 | 34 | 5.75 | 0.29 (0.21-0.41) | <0.001 | 0.51 (0.36-0.72) | <0.001 |
| **Without cardiovascular disease** | | | | | | | |
| Sedentary | 15494 | 298 | 5.73 | Reference |  | Reference |  |
| 1-499 MET-min/week | 10838 | 124 | 3.34 | 0.58 (0.47-0.72) | <0.001 | 0.74 (0.60-0.91) | 0.005 |
| 500-999 MET-min/week | 10782 | 88 | 2.41 | 0.42 (0.33-0.53) | <0.001 | 0.56 (0.44-0.72) | <0.001 |
| 1000-1499 MET-min/week | 3321 | 21 | 1.83 | 0.32 (0.20-0.49) | <0.001 | 0.47 (0.30-0.73) | <0.001 |
| ≥1500 MET-min/week | 3917 | 28 | 2.09 | 0.36 (0.25-0.53) | <0.001 | 0.52 (0.35-0.77) | 0.001 |

* Adjusted for age, sex, body mass index, hypertension, diabetes mellitus, dyslipidemia, chronic kidney disease, chronic obstructive pulmonary disease, malignancy, smoking, alcohol, osteoporosis, hospital frailty risk score, Charlson comorbidity index score

MET, metabolic equivalent task; HR, hazard ratio; CI, confidence interval

**Supplementary Table 6.** Leisure-time physical activity and risk of non-cardiovascular death stratified according to the presence of cardiovascular disease

|  | **Patients** | **Deaths** | **Deaths, /1000PYR** | **Unadjusted HR (95% CI)** | **P value** | **Adjusted HR (95% CI)** | **P value** |
| --- | --- | --- | --- | --- | --- | --- | --- |
| **With cardiovascular disease** | | | | | | | |
| Sedentary | 9760 | 1161 | 40.03 | Reference |  | Reference |  |
| 1-499 MET-min/week | 5721 | 393 | 21.67 | 0.54 (0.48-0.61) | <0.001 | 0.69 (0.62-0.78) | <0.001 |
| 500-999 MET-min/week | 5178 | 331 | 20.13 | 0.50 (0.44-0.57) | <0.001 | 0.70 (0.61-0.79) | <0.001 |
| 1000-1499 MET-min/week | 1444 | 59 | 12.50 | 0.31 (0.24-0.40) | <0.001 | 0.47 (0.36-0.61) | <0.001 |
| ≥1500 MET-min/week | 1768 | 78 | 13.36 | 0.33 (0.26-0.42) | <0.001 | 0.47 (0.38-0.59) | <0.001 |
| **Without cardiovascular disease** | | | | | | | |
| Sedentary | 15494 | 1269 | 24.72 | Reference |  | Reference |  |
| 1-499 MET-min/week | 10838 | 544 | 14.84 | 0.60 (0.54-0.66) | <0.001 | 0.75 (0.68-0.84) | <0.001 |
| 500-999 MET-min/week | 10782 | 522 | 14.46 | 0.59 (0.53-0.65) | <0.001 | 0.75 (0.68-0.83) | <0.001 |
| 1000-1499 MET-min/week | 3321 | 125 | 11.00 | 0.44 (0.37-0.53) | <0.001 | 0.62 (0.51-0.74) | <0.001 |
| ≥1500 MET-min/week | 3917 | 176 | 13.28 | 0.54 (0.46-0.63) | <0.001 | 0.70 (0.59-0.82) | <0.001 |

* Adjusted for age, sex, body mass index, hypertension, diabetes mellitus, dyslipidemia, chronic kidney disease, chronic obstructive pulmonary disease, malignancy, smoking, alcohol, osteoporosis, hospital frailty risk score, Charlson comorbidity index score

MET, metabolic equivalent task; HR, hazard ratio; CI, confidence interval

**Supplementary Table 7.** Leisure-time physical activity and the risk of all-cause mortality according to subgroups in patients with cardiovascular disease

| **Subgroup** | **Fully adjusted hazard ratio (95% confidence interval)** | | | | | |
| --- | --- | --- | --- | --- | --- | --- |
|  | **Leisure-time physical activity, MET-min/week** | | | | | |
|  | **Sedentary** | **1-499** | **500-999** | **1000-1499** | **≥1500** | **P for interaction** |
| **Age** | | | | | | 0.748 |
| <75 | 1.00 (Ref.) | 0.79 (0.67-0.94) | 0.68 (0.57-0.80) | 0.62 (0.47-0.82) | 0.46 (0.35-0.61) |  |
| ≥75 | 1.00 (Ref.) | 0.64 (0.56-0.72) | 0.71 (0.62-0.81) | 0.52 (0.38-0.71) | 0.53 (0.40-0.69) |  |
| **Sex** | | | | | | 0.077 |
| Male | 1.00 (Ref.) | 0.62 (0.54-0.71) | 0.59 (0.51-0.68) | 0.49 (0.36-0.65) | 0.49 (0.38-0.63) |  |
| Female | 1.00 (Ref.) | 0.69 (0.60-0.78) | 0.68 (0.58-0.79) | 0.62 (0.45-0.86) | 0.37 (0.25-0.56) |  |
| **Body mass index** | | | | | | 0.313 |
| <25 | 1.00 (Ref.) | 0.70 (0.63-0.79) | 0.69 (0.61-0.78) | 0.53 (0.42-0.68) | 0.48 (0.38-0.60) |  |
| ≥25 | 1.00 (Ref.) | 0.66 (0.54-0.81) | 0.71 (0.57-0.88) | 0.66 (0.45-0.96) | 0.54 (0.38-0.78) |  |
| **Smoking** | | | | | | 0.828 |
| No | 1.00 (Ref.) | 0.69 (0.61-0.77) | 0.66 (0.58-0.75) | 0.58 (0.45-0.75) | 0.36 (0.27-0.48) |  |
| Yes | 1.00 (Ref.) | 0.71 (0.58-0.88) | 0.79 (0.65-0.95) | 0.52 (0.36-0.74) | 0.68 (0.52-0.89) |  |
| **Alcohol** | | | | | | 0.185 |
| No | 1.00 (Ref.) | 0.67 (0.61-0.75) | 0.67 (0.60-0.76) | 0.59 (0.47-0.74) | 0.46 (0.36-0.57) |  |
| Yes | 1.00 (Ref.) | 0.83 (0.62-1.10) | 0.82 (0.62-1.08) | 0.47 (0.28-0.77) | 0.61 (0.41-0.90) |  |
| **Hypertension** | | | | | | 0.162 |
| No | 1.00 (Ref.) | 0.60 (0.46-0.78) | 0.56 (0.42-0.74) | 0.39 (0.21-0.75) | 0.35 (0.19-0.63) |  |
| Yes | 1.00 (Ref.) | 0.71 (0.63-0.79) | 0.71 (0.64-0.80) | 0.59 (0.47-0.73) | 0.50 (0.41-0.62) |  |
| **Diabetes** | | | | | | 0.823 |
| No | 1.00 (Ref.) | 0.71 (0.63-0.80) | 0.72 (0.63-0.82) | 0.62 (0.48-0.80) | 0.51 (0.40-0.65) |  |
| Yes | 1.00 (Ref.) | 0.66 (0.56-0.78) | 0.64 (0.53-0.76) | 0.48 (0.34-0.66) | 0.44 (0.32-0.61) |  |
| **Dyslipidemia** | | | | | | 0.050 |
| No | 1.00 (Ref.) | 0.68 (0.57-0.80) | 0.78 (0.65-0.93) | 0.64 (0.45-0.90) | 0.62 (0.46-0.84) |  |
| Yes | 1.00 (Ref.) | 0.70 (0.62-0.79) | 0.64 (0.56-0.74) | 0.52 (0.40-0.67) | 0.43 (0.33-0.55) |  |
| **Chronic kidney disease** | | | | | | 0.691 |
| No | 1.00 (Ref.) | 0.68 (0.61-0.76) | 0.67 (0.60-0.75) | 0.56 (0.45-0.69) | 0.49 (0.40-0.60) |  |
| Yes | 1.00 (Ref.) | 0.84 (0.60-1.19) | 0.99 (0.71-1.39) | 0.53 (0.23-1.21) | 0.40 (0.18-0.87) |  |
| **Malignancy** | | | | | | 0.984 |
| No | 1.00 (Ref.) | 0.69 (0.61-0.77) | 0.72 (0.64-0.81) | 0.60 (0.47-0.75) | 0.48 (0.38-0.60) |  |
| Yes | 1.00 (Ref.) | 0.68 (0.56-0.82) | 0.60 (0.48-0.74) | 0.42 (0.27-0.67) | 0.47 (0.33-0.67) |  |
| **Osteoporosis** | | | | | | 0.118 |
| No | 1.00 (Ref.) | 0.70 (0.61-0.81) | 0.67 (0.58-0.77) | 0.57 (0.44-0.74) | 0.54 (0.43-0.68) |  |
| Yes | 1.00 (Ref.) | 0.68 (0.58-0.78) | 0.72 (0.62-0.85) | 0.53 (0.38-0.75) | 0.37 (0.25-0.54) |  |

* Adjusted for age, sex, body mass index, hypertension, diabetes mellitus, dyslipidemia, chronic kidney disease, chronic obstructive pulmonary disease, malignancy, smoking, alcohol, osteoporosis, hospital frailty risk score, Charlson comorbidity index score

MET, metabolic equivalent task
