# Supplementary material for: Impact of Physical Activity on All-Cause Mortality According to Specific Cardiovascular Disease
Source: Front Cardiovasc Med. 2022 Feb 4;9:811058. doi: 10.3389/fcvm.2022.811058 (PMC8855984; doi:10.3389/fcvm.2022.811058)

Supplementary Material

Supplementary Figure 1. Flow chart of study population enrollment and analysis

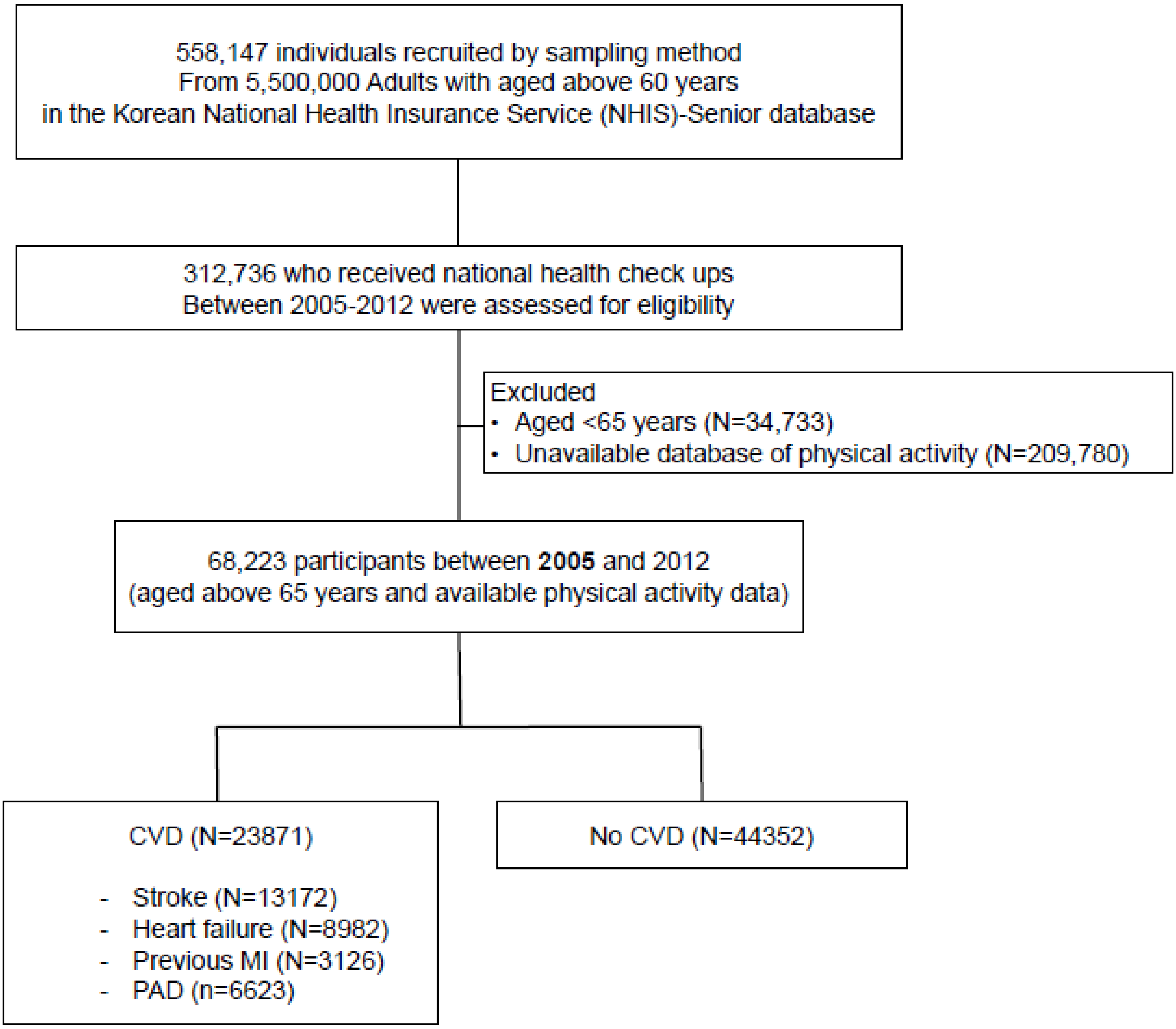

Supplementary Figure 2. Physical activity level of participants with specific cardiovascular disease

(A) Stroke, (B) heart failure, (C) previous myocardial infarction (MI), (D) peripheral artery disease (PAD)

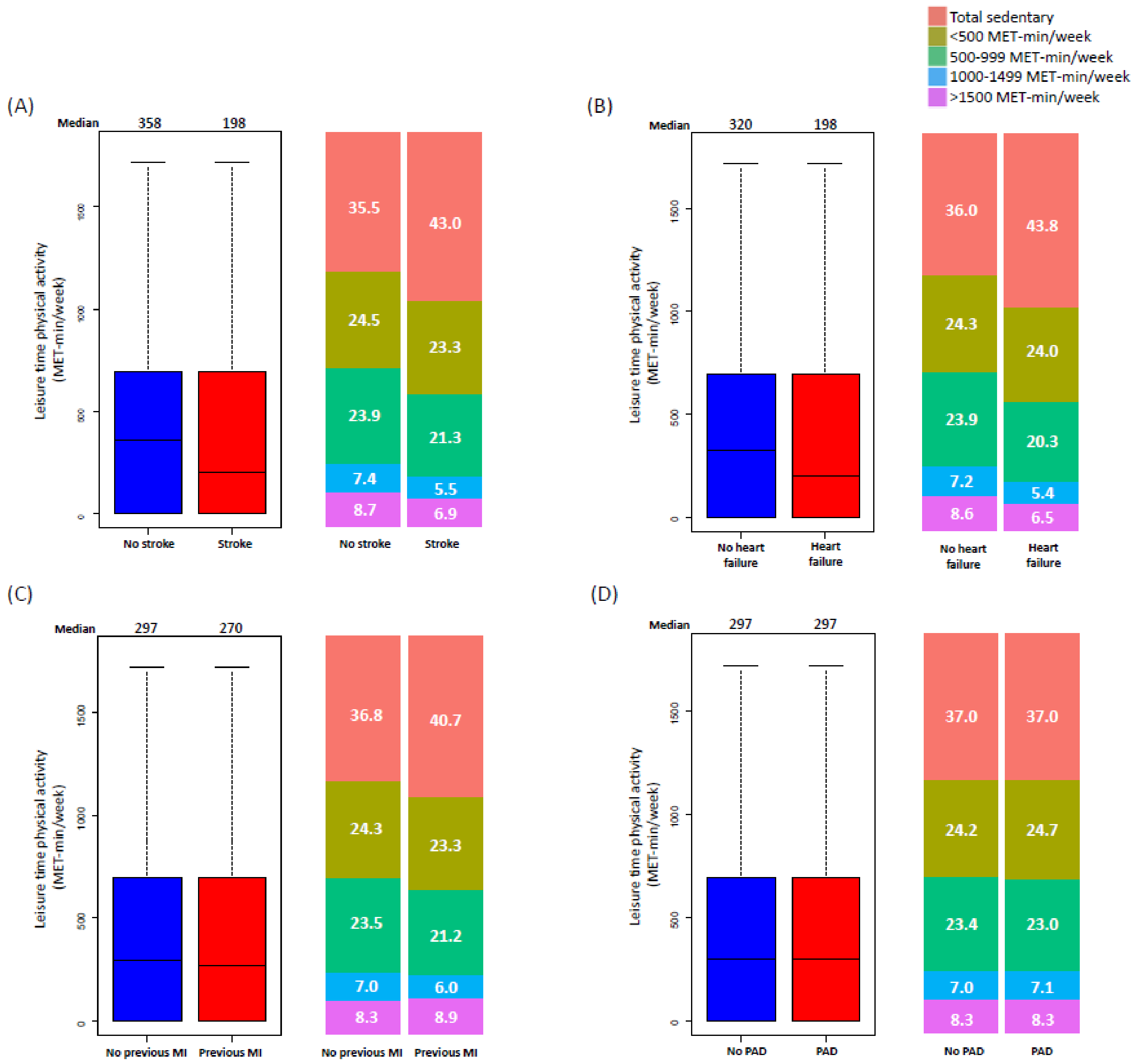

Supplement: Supplementary file 2 [file Image_1.PDF]
